# Late-onset myopathy of the posterior calf muscles mimicking Miyoshi myopathy unrelated to dysferlin mutation: a case report

**DOI:** 10.1186/1752-1947-6-345

**Published:** 2012-10-10

**Authors:** Clemens Neusch, Tanja Kuhlmann, Wolfram Kress, Christiane Schneider-Gold

**Affiliations:** 1 Department of Neurology, University of Ulm, Ulm, 89081, Germany; 2 Institute of Neuropathology, University Hospital Münster, Münster, 48149, Germany; 3 Institute of Human Genetics, University of Würzburg, Biozentrum, Am Hubland, Würzburg, 97074, Germany; 4 Department of Neurology, University of Bochum, St Josef Hospital, Bochum, 44791, Germany

## Abstract

**Introduction:**

Miyoshi myopathy, a type of distal myopathy with predominant involvement of the posterior calf muscles, has been assigned to mutations in the dysferlin gene. However, many of the late-onset limb-girdle and distal myopathies that resemble dysferlinopathy or Miyoshi myopathy remain unclassified, even after extensive immunohistological and genetic analysis.

**Case presentation:**

We report the case of a 59-year-old Caucasian man with distal myopathy and exercise-induced myalgia, preferentially of the leg muscles, closely resembling the Miyoshi phenotype. Magnetic resonance imaging of his calf muscles showed typical fatty replacement of the medial heads of the gastrocnemius muscles and soleus muscles, with progression to the adductor longus muscles over a time course of two years. However, genetic analysis revealed that the phenotype of our patient was not related to a mutation in the dysferlin gene but to a novel homozygous splice mutation in the anoctamin 5 gene. Mutations in the anoctamin 5 gene have so far been identified only in some cases of limb-girdle and distal myopathy. Mutations in the anoctamin 5 gene have been assigned to limb-girdle muscular dystrophy type 2L, while distal Miyoshi-like phenotypes have been classified as Miyoshi myopathy type 3.

**Conclusion:**

The case presented in this report further strengthens the underlying genetic heterogeneity in Miyoshi myopathy-like phenotypes and adds another family to non-dysferlin, Miyoshi myopathy type 3 of late-onset. Furthermore, our case supports the recent observation that anoctamin 5 mutations are a primary cause of distal non-dysferlin myopathies. Therefore, given the increasing number of anoctamin 5 mutations in Miyoshi-like phenotypes, genetic analysis should include an anoctamin 5 screen in late-onset limb-girdle and distal myopathies.

## Introduction

Miyoshi myopathy (MM) is caused by autosomal recessive mutations in the human dysferlin gene [1,2]. In MM, involvement of the gastrocnemius and soleus muscles with difficulties in standing on tip toes is characteristic. Clinical signs usually occur in the late teens and mostly before the age of 30 years. According to its clinical and histopathological presentation, MM has been categorized as distal myopathy with typical findings of muscular dystrophy [3,4].

Late-onset forms of distal muscular dystrophies with features of MM have been described. These rare cases with disease onset after the age of 30 years [5] have not only been attributed to mutations in the dysferlin gene but have also been described in patients with normal dysferlin expression [5]. These reports have suggested other genetic defects causing the MM. Recently, recessive mutations in the anoctamin 5 (ANO5) gene were identified as the cause of predominantly asymmetric limb-girdle muscular dystrophy (LGMD type 2L) as well as distal myopathies of the MM phenotype (MMD3) [6,7]. The protein ANO5 is coded by exons 1 to 22 of the *ANO5/TMEM16E* gene located on chromosome 11p14.3. The ANO5 gene encodes a putative calcium-related chloride channel; however, the precise function of ANO5 is unknown.

## Case presentation

A 59-year-old Caucasian man was admitted to hospital with slowly progressive muscle weakness and wasting of his posterior calf muscles. Neurological examination revealed normal cranial nerve function and intellectual status. Manual muscle testing showed weakness of Medical Research Council (MRC) grade 4+ out of 5 in his posterior calf muscles and marginal weakness MRC grade 5- out of 5 in his anterior calf muscles. His deep tendon reflexes were symmetrically preserved. His family history was suggestive for an autosomal recessive trait. Serum creatine kinase values were normal in our patient’s two children and one sibling.

**Figure 1:**
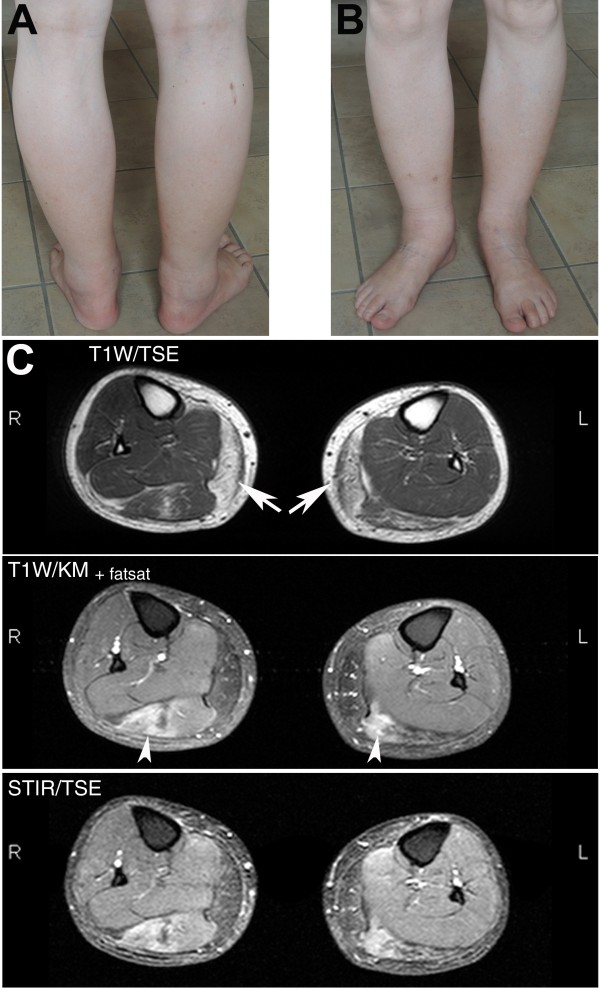
** Photographs and magnetic resonance imaging scans of the patient’s legs. (A**,**B)** Photographs taken of patient’s legs. Note muscle wasting that predominantly affects posterior calf muscles (**A**). (**C**) Magnetic resonance images of both calves (top: T1-weighted image; middle: T1-weighted image, contrast enhanced, fat suppressed; bottom: Short T1 inversion recovery (STIR) images). Fatty degeneration of the medial heads of the gastrocnemius muscles (arrows). Additional areas of edema and contrast enhancement in the lateral head of the gastrocnemius muscle indicate muscle inflammation (arrowhead).

On magnetic resonance imaging, T1-weighted turbo spin echo and short T1 inversion recovery (fat-suppressed) sequences revealed selective atrophy and fatty replacement of the medial heads of his gastrocnemius muscles and soleus muscles (Figure 1). In follow-up magnetic resonance imaging two years later, this was also visible in his adductor longus muscles (not shown). Contrast-enhanced images showed areas of fatty degeneration next to areas with contrast enhancement and edema in the lateral head of his gastrocnemius muscles and the proximal part of his soleus muscles.

Needle electromyography of both gastrocnemius muscles showed sparse positive sharp waves. At voluntary effort, early recruitment of short and small motor unit action potentials compatible with a myopathic pattern was observed. Nerve conduction studies revealed normal sensory nerve action potentials and conduction velocities of his right median and sural nerve as well as normal compound muscle action potential amplitudes and nerve conduction velocities in his right median and tibial nerve. His serum creatine kinase level was markedly elevated up to 1700U/L (normal, less than 270U/L); lactate dehydrogenase, 264U/L (normal, less than 232U/L); alanine aminotransferase, 53U/L (normal, less than 35U/L); and aspartate aminotransferase, 67U/L (normal, less than 45U/L). His gamma-glutamyl-transpeptidase level was normal.

**Figure 2:**
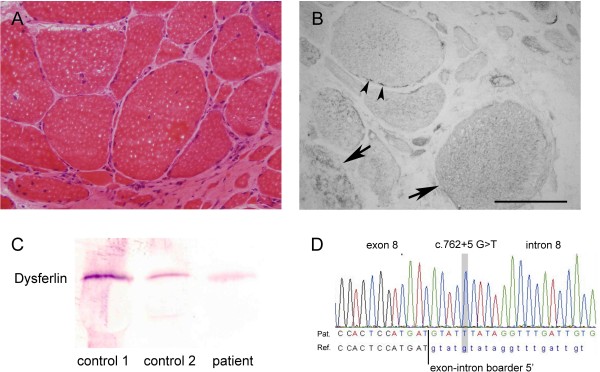
** Immunohistochemistry, immunoblotting and molecular genetic analysis.** (**A**) Micrograph of the muscle biopsy of the right gastrocnemius muscle showing markedly increased variation in fiber size, fiber splitting, increased number of internal nuclei, some very atrophic fibers, and proliferation of adipose and fibrous tissue (hematoxylin and eosin staining, ×100). (**B**) Dysferlin labeling of the muscle specimen. Arrows point to fibers with diffuse and partly granular cytoplasmic dysferlin accumulation. Arrowheads indicate exemplary fibers with remaining patchy dysferlin signal of the fiber sarcolemma while most fibers completely lack sarcolemmal dysferlin. Bar = 100μM. (**C**) Immunoblot on muscle lysates from the patient and two controls showing comparable protein expression levels. (**D**) Sequencing electropherogram of partial *anoctamin 5* exon 8 and intron 8 sequences reveals a novel splice-site mutation at intron 8 that may result in skipping of exon 9.

Hematoxylin and eosin staining of serial frozen sections of his gastrocnemius muscle revealed characteristic myopathic changes including marked variation in fiber size, centrally localized nuclei, and necrotic and regenerating fibers. Furthermore, moderate endomysial fibrosis as well as some small perivascular inflammatory infiltrates was observed (Figure 2). Immunohistochemical stainings were performed with antibodies against dystrophin (Dys1 and Dys3), γ- and δ-sarcoglycan, vimentin, desmin, dysferlin (all from Novocastra, Berlin, Germany), Dys2, spectrin II, utrophin, α- and β-sarcoglycan, α- and β-dystroglycan (all from Medac, Wedel, Germany), NCL-merosin (Chemicon, Temecula, CA, USA), laminin α5 and laminin β1 (Invitrogen, Grand Island, NY, USA), actin (Dako, Glostrup, Denmark), and caveolin 3 (Dianova, Hamburg, Germany). Dysferlin immunoreactivity was markedly reduced at muscle fiber membranes but diffusely increased in the cytoplasm of many fibers (Figure 2B). Utrophin expression was slightly upregulated (data not shown). Immunoblot analysis was performed using a mini-Protean II Western blotting system (BioRad, Herts, UK). Immunoblotting analysis did not reveal any significant reduction of dysferlin (Figure 2C), dystrophin or calpain 3 (data not shown).

Molecular genetic analyses were performed after obtaining informed consent from our patient. DNA extraction and direct sequencing of the complete dysferlin gene was done as described previously [8]. Molecular genetic analysis of fukutin-related protein, β-sarcoglycan, calpain-3 and caveolin-3, a putative interaction and membrane targeting partner of dysferlin, and multiplex ligation-dependent probe amplification analysis of the dystrophin gene (MRC Holland, Amsterdam, The Netherlands) ruled out underlying mutations with high probability. Finally, a novel homozygous splice mutation, c.762 + 5G > T, in the ANO5 gene was identified after direct Sanger sequencing of all coding exons of *ANO5* (Reference sequence NM_ 213599.2). Splice prediction programs (Alamut mutation prediction program, version 2.1, Interactive Biosoftware, Rouen, France) showed a loss of the splice donor site. Whilst we could not determine the impact of this mutation at the protein level it is suggested that exon 9 could be skipped due to the identified splice donor site mutation in intron 8.

## Discussion

We report an MM-like phenotype caused by a homozygous IVS 8, c.762 + 5G > T mutation in the ANO5 gene. In some previous cases of late-onset distal myopathies with predominant involvement of the posterior calf muscles, immunoblotting showed reduced dysferlin levels, although no dysferlin gene mutation could be found [9]. Other groups described patients with early-onset and late-onset distal myopathies preferentially involving the calf muscles who had normal dysferlin expression [5]. Thus it became clear that the phenotype of distal myopathy with predominant involvement of the posterior calf muscles is not restricted to MMD1 (the MM type of dysferlinopathy). So far, only a few patients with the Miyoshi-like phenotype caused by an *ANO5* mutation have been described [6,7]. The manifestation of a limb-girdle type of myopathy seems to be much more frequent in *ANO5* mutations (LGMD type 2L) [6,10]. In most cases, asymmetric involvement of proximal or distal muscles was observed. In contrast with these early observations in *ANO5* mutations, our patient showed symmetric weakness and wasting of calf muscles which is the clinical hallmark of MMD3. As in our case, myalgia appears to be a typical symptom in *ANO5*-related myopathies [6].

## Conclusion

We present a case of a patient with late-onset, non-dysferlin MM with an underlying, novel splice mutation in *ANO5*. Our observation enlarges the spectrum of MM-like myopathies caused by an *ANO5* mutation and further strengthens the underlying genetic heterogeneity of the MM-like muscle diseases, especially in late-onset phenotypes. The mutation described here is associated with reduced sarcolemmal dysferlin and granular cytoplasmic dysferlin accumulation on immunohistochemistry while dysferlin immunoblot shows equal protein levels to controls. It remains to be proven whether other types of anoctaminopathies also show this pattern of dysferlin expression. For the moment, our findings imply that dysferlin-staining abnormalities, as in our case, could give a diagnostic hint to look for an *ANO5* mutation and that some pathomechanisms could be linked to altered dysferlin distribution.

## Consent

Written informed consent was obtained from the patient for publication of this case report and any accompanying images. A copy of the written consent is available for review by the Editor-in-Chief of this journal.

## Competing interests

The authors declare that they have no competing interests.

## Authors’ contributions

CN participated in the examination of the patient, the design and coordination of the study and drafted the manuscript. TK performed the immunohistochemistry and analyzed the biopsy material and the immunolabeling. WK participated in the genetic analysis and in the immunoblotting and analyzed this set of data. CSG participated in the examination of the patient, the design and coordination and helped to draft and revise the manuscript for important intellectual content. All authors read and approved the final manuscript.
